# Stochastic partial differential fluid equations as a diffusive limit of deterministic Lagrangian multi-time dynamics

**DOI:** 10.1098/rspa.2017.0388

**Published:** 2017-09-20

**Authors:** C. J. Cotter, G. A. Gottwald, D. D. Holm

**Affiliations:** 1 Department of Mathematics, Imperial College, London, UK; 2 School of Mathematics and Statistics, University of Sydney, Sydney, Australia

**Keywords:** geometric mechanics, stochastic fluid models, stochastic processes, multi-scale fluid dynamics, symmetry reduced variational principles, homogenization

## Abstract

In Holm (Holm 2015 *Proc. R. Soc. A* **471**, 20140963. (doi:10.1098/rspa.2014.0963)), stochastic fluid equations were derived by employing a variational principle with an assumed stochastic Lagrangian particle dynamics. Here we show that the same stochastic Lagrangian dynamics naturally arises in a multi-scale decomposition of the deterministic Lagrangian flow map into a slow large-scale mean and a rapidly fluctuating small-scale map. We employ homogenization theory to derive effective slow stochastic particle dynamics for the resolved mean part, thereby obtaining stochastic fluid partial equations in the Eulerian formulation. To justify the application of rigorous homogenization theory, we assume mildly chaotic fast small-scale dynamics, as well as a centring condition. The latter requires that the mean of the fluctuating deviations is small, when pulled back to the mean flow.

## 1. Introduction

When studying complex turbulent flows or astrophysical and geophysical fluids, in which physical processes occur over a wide range of spatial and temporal scales, we are faced with the inevitable problem that our limited computational resources will eventually force us to under-represent processes that occur below certain temporal and spatial scales. Processes with spatial scales smaller than the grid scale and with temporal scales below the applied discrete time step simply cannot be resolved. However, these unresolved processes may be energetically important and could have a non-trivial impact on the resolved scales. For example, in the study of mid-latitude weather, one is generally interested in the dynamics of the synoptic weather systems such as high- and low-pressure fields with spatial scales of several hundred kilometres and time scales of several days. Numerical simulations must choose a discretization to resolve those processes. The atmosphere, however, also supports gravity waves, which are the buoyancy oscillations of the stratification surfaces with spatial scales of hundred metres and time scales of several minutes to hours. These unresolved gravity waves play a crucial role in vertical momentum transport; so their omission in a numerical simulation would lead to a misrepresentation of this transport. Similarly, ocean current models do not resolve meso-scale eddies which transport momentum, heat and salinity over large spatial scales. Scientists therefore search for methods to parametrize the influence of these unresolved processes.

Recently, stochastic partial differential fluid equations have been proposed to model the influence of unresolved scales on the resolved scales of interest [1–5]. These novel approaches introduce stochasticity into the flow map for the Lagrangian particle trajectories, then the noise in the Lagrange-to-Euler map produces a random Eulerian vector field. This approach results in an interesting form of multiplicative noise in the Eulerian stochastic partial differential fluid equation (SPDE). In the SPDE, the gradient of the noise and its magnitude multiply, respectively, the solution and the gradient of the solution. Thus, the effects of the noise depend on spatial gradients of both the noise and the solution. In [1,3–5], the transformation from the Lagrangian to the Eulerian fluid formulation was achieved via a particular version of the Reynold's transport theorem that preserved conservation of energy.

In [2], a variational principle was employed in the derivation of the SPDE for fluids. The same model was later derived in [6] from Newton's second Law of Motion and a different version of the Reynold's transport theorem, which included the transformation of the coordinate basis under the Lagrange-to-Euler map. Instead of preserving the energy, the model derived in [2,6] has the property of preserving Kelvin's circulation theorem, while introducing a stochastic advecting velocity for the Kelvin loop, which transports the line elements of the Kelvin loop along stochastic Lagrangian trajectories.

The stochastic Eulerian random field associated with this advecting velocity vector field was prescribed in [2,6]. Here, we will motivate the stochastic advection of fluid particles by using homogenization techniques [7]. In particular, we will show that the same form of stochasticity as in [2,6] naturally arises, upon splitting the Lagrangian fluid flow map into the composition of mean and fluctuating maps that is standard for generalized Lagrangian mean theories [8–11]. In particular, when the time scale of the fluctuating map is sufficiently rapid in comparison to the slow mean field dynamics, and when the fast dynamics is sufficiently chaotic, its integrated effect on the slow mean field dynamics is an effective stochastic term, whose variance is determined via the autocorrelation function of the fast fluctuating flow map.

The paper is organized as follows. Section 2 briefly summarizes the variational stochastic fluid dynamics introduced in Holm [2]. Section 3 then constructs a deterministic multi-scale Lagrangian particle system. In §4, we employ homogenization to derive the stochastic noise in the Lagrangian dynamics, assumed in the variational stochastic fluid approach, from the deterministic multi-scale system. We conclude in §5 with a summary and discussion.

## 2. Variational stochastic fluid equations

### (a) Variational principles for deterministic fluids

Governing equations for a variety of ideal fluid dynamics theories can be derived within a variational framework as the Euler–Poincaré equations associated with the following variational principle with the action [12]

2.1 $$S(\text{u},\text{p},\text{q})=\int \ell (\text{u},a)\;dt+\langle \text{p},\partial_{t}\text{q}-\text{u}(\text{q},t)\rangle \;dt,$$

where, **q**(**l**,*t*)=*g*(*t*)**l** is the Lagrangian trajectory for the time-dependent fluid particle flow map *g*(*t*), with *g*(0)=*Id*. In this paper, we will denote explicit time dependence with either parentheses, such as *g*(*t*), or with subscript *t*, such as as *g*_*t*_; while as usual ∂_*t*_ will denote partial time derivative and a dot a total time derivative. For example, the fluid particle flow map **q**(**l**,*t*)=*g*(*t*)**l**=*g*_*t*_**l** provides the spatial location of the particle with label **l** at time *t*. Accordingly, the time derivative along the Lagrangian particle trajectory **q**(**l**,*t*) satisfies the defining relation for the Eulerian velocity, **u**_*t*_, given by

2.2 $$\dot{\text{q}}(\text{l},t)={\dot{g}}_{t}\text{l}=({\dot{g}}_{t}g_{t}^{-1})g_{t}\text{l}={\text{u}}_{t}\circ g_{t}\text{l}:=\text{u}(\text{q}(\text{l},t),t).$$

Thus, in this notation, ${\text{u}}_{t}={\dot{g}}_{t}g_{t}^{-1}$ is an Eulerian time-dependent velocity vector field, $L(g_{t},{\dot{g}}_{t},a_{0})=L(g_{0},{\dot{g}}_{t}g_{t}^{-1},a_{0}g_{t}^{-1})=\ell ({\text{u}}_{t},a_{t})$ is the reduced Lagrangian, in which $a_{t}:=a_{0}\circ g_{t}^{-1}$ represents a set of advected quantities; such as advected tracers, $\theta =\theta_{0}\circ g_{t}^{-1}$, or advected densities, $\rho \;d^{3}x=(\rho_{0}\;d^{3}l)\circ g_{t}^{-1}$.

Relation (2.2) is encoded as a constraint in the Clebsch variational formulation in (2.1) with the Lagrange multiplier **p**(**l**,*t*), via the inner product

2.3 $$\langle \text{p},\dot{\text{q}}\rangle =\int_{\Omega }\text{p}(\text{l},t)\cdot \dot{\text{q}}(\text{l},t)\;d^{n}\text{l},$$

where *n* is the dimension of the domain *Ω*. The extremal points of the constrained, reduced action in (2.1) then lead to the Euler–Poincaré equations

2.4 $$\frac{\partial }{\partial t}\frac{\delta \ell }{\delta \text{u}}+{\mathrm{ad}}_{\text{u}}^{*}\frac{\delta \ell }{\delta \text{u}}=a⋄\frac{\delta \ell }{\delta a},$$

where one defines ad*_u_(**v**⋅d**x**⊗d^*n*^*x*)=*L*_**u**_(**v**⋅d**x**⊗d^*n*^*x*), which has components

2.5 $${\mathrm{ad}}_{\text{u}}^{*}\text{v}=\nabla \cdot (\text{u}⊗\text{v})+{\left(\nabla \text{u}\right)}^{T}\cdot \text{v}=L_{\text{u}}\text{v}$$

and the diamond operator (⋄) is defined as

2.6 $$\int_{\Omega }\text{w}\cdot a⋄\phi \;d^{n}x=\int_{\Omega }\phi L_{\text{w}}a\;d^{n}x,$$

for all test functions *ϕ* and **w** (satisfying the boundary conditions **w**⋅**n**=0 on ∂*Ω*).

In (2.5), *L*_**w**_*a* denotes the advection law for the advected quantity in question. Since advected fluid quantities satisfy *a*_0_=*a*_*t*_°*g*_*t*_=*a*(**q**(**l**,*t*),*t*)=:g*_t_*a*(**x**,*t*), we have the advection law

2.7 $$\begin{array}{rl} \frac{da_{0}}{dt}=0 & \;=\frac{d}{dt}(g_{t}^{*}a(\text{x},t))=g_{t}^{*}((\partial_{t}+L_{\text{u}})a_{t})=\frac{da_{t}}{dt}\;\text{along}\;\dot{\text{q}}(\text{l},t)=\text{u}(\text{x},t){|}_{\text{x}=\text{q}(\text{l},t)}\\ & \;={\left[\partial_{t}a_{t}+\dot{\text{q}}(\text{l},t)\cdot \nabla a_{t}\right]}_{\text{x}=\text{q}(\text{l},t)}={\left[\partial_{t}a_{t}+\text{u}(\text{x},t)\cdot \nabla a_{t}\right]}_{\text{x}=\text{q}(\text{l},t)}. \end{array}$$

For example, if *a*=*ρ* d^*n*^*x* is a density, then g*_t_*a*=g*_t_(*ρ* d^*n*^*x*)=[*ρ*(**x**,*t*) d^*n*^*x*]_**x**=**q**(**l**,*t*)_. Thus, the advection law for a density satisfies the continuity equation in Eulerian coordinates, since in this case the previous formula becomes

2.8 $$0=\frac{d}{dt}{\left[\rho (\text{x},t)\;d^{n}x\right]}_{\text{x}=\text{q}(\text{l},t)}={\left[(\partial_{t}\rho +\text{u}\cdot \nabla \rho +\rho \nabla \cdot \text{u})\;d^{n}x\right]}_{\text{x}=\text{q}(\text{l},t)}.$$

Consequently, for a density *ρ* d^*n*^*x* and a scalar function *ϕ*, we find ρ⋄ϕ=−ρ∇ϕ, since

2.9 $$\int_{\Omega }\text{w}\cdot \rho ⋄\phi \;d^{n}x=\int_{\Omega }\phi L_{\text{w}}\rho \;d^{n}x=\int_{\Omega }\phi \nabla \cdot (\text{w}\rho )\;d^{n}x=-\int_{\Omega }\text{w}\cdot (\rho \nabla \phi )\;d^{n}x.$$

It is slightly more complicated to prove equation (2.5). However, the same rules apply; so

2.10 $$\begin{array}{rl} g_{t}^{*}(L_{\text{u}}(\text{v}\cdot d\text{x}⊗d^{n}x)) & \;=\frac{d}{dt}(g_{t}^{*}(\text{v}(\text{x})\cdot d\text{x}⊗d^{n}x))=\frac{d}{dt}{\left[\text{v}(\text{x})\cdot d\text{x}⊗d^{n}x\right]}_{\text{x}=\text{q}(\text{l},t)}\\ & \;={\left[((\text{u}\cdot \nabla )\text{v}+v_{j}\nabla u^{j}+\text{v}(\nabla \cdot \text{u}))\cdot d\text{x}⊗d^{n}x\right]}_{\text{x}=\text{q}(\text{l},t)}\\ & \;={\left[(\nabla \cdot (\text{u}⊗\text{v})+{\left(\nabla \text{u}\right)}^{T}\cdot \text{v})\cdot d\text{x}⊗d^{n}x\right]}_{\text{x}=\text{q}(\text{l},t)}\\ & \;=g_{t}^{*}[(\nabla \cdot (\text{u}⊗\text{v})+{\left(\nabla \text{u}\right)}^{T}\cdot \text{v})\cdot d\text{x}⊗d^{n}x]. \end{array}$$

The proof of equation (2.5) then follows, because in this case we have the identity,

2.11 $$L_{\text{u}}(\text{v}\cdot d\text{x}⊗d^{n}x)={\mathrm{ad}}_{\text{u}}^{*}(\text{v}\cdot d\text{x}⊗d^{n}x).$$

Different choices of *a* and ℓ lead to a huge range of equation systems in fluid dynamics, including magnetohydrodynamics, geophysical fluid dynamics and complex fluids, all formulated in terms of their transformation properties under smooth invertible flow maps; see the examples in [12]. The possibility to eliminate **p** and **q** from the equations is a result of the particle relabelling transformation properties of the action *S*, which leads to Kelvin's circulation theorem, expressed as [12]

2.12 $$\frac{d}{dt}\oint_{C(t)}\frac{1}{\rho }\frac{\delta \ell }{\delta \text{u}}\cdot d\text{x}=\oint_{C(t)}\frac{1}{\rho }\left(a⋄\frac{\delta \ell }{\delta a}\right)\cdot d\text{x},$$

where *C*(*t*) is an arbitrary closed loop which is advected by the velocity **u**. This leads, for example, to Lagrangian conserved potential vorticity, in particular instances of the advected quantity *a*. The equations also have a conserved energy provided ℓ does not depend explicitly on time, which arises from Noether's theorem for variational principles that are invariant under time translations.

For example, the reduced Lagrangian for the incompressible Euler equations is

2.13 $$\ell [\text{u},\rho ]=\int_{\Omega }\frac{1}{2}{\rho |\text{u}|}^{2}+p(\rho -\rho_{0})\;d^{n}x,$$

where *p* is a Lagrange multiplier (which will turn out to be the pressure) enforcing constant density *ρ*=*ρ*_0_. Then, the Euler–Poincaré equations with advected quantities become

2.14 $$\begin{array}{rl} & \partial_{t}(\rho \text{u})+\nabla \cdot (\text{u}⊗\rho \text{u})+{\left(\nabla \text{u}\right)}^{T}\cdot \rho \text{u}=\rho \nabla \left(-p+\frac{|\text{u}|}{2}\right), \end{array}$$

2.15 $$\begin{array}{rl} & \rho_{t}+\nabla \cdot (\rho \text{u})=0 \end{array}$$

2.16 $$\begin{array}{rl} \text{and}\; & \rho =\rho_{0}. \end{array}$$

When *ρ*_0_ is a constant, these equations simplify to

2.17 $$\partial_{t}\text{u}+\text{u}\cdot \nabla \text{u}=-\nabla p\;\text{and}\;\nabla \cdot \text{u}=0.$$

### (b) Variational principles for stochastic fluids

In [2,6], this framework was extended to include stochastic parametrizations of unresolved dynamics, by replacing the deterministic equation (2.2) with a stochastic equation

2.18 $$d\text{q}=\text{u}(\text{q},t)\;dt+\sum_{i=1}^{m}\xi_{i}(\text{q})\circ dW_{i},$$

where ***ξ***_*i*_(**q**) are time-independent vector fields, and *W*_*i*_ are independent stochastic Brownian paths. Inserting the stochastic vector field Ansatz (2.18) into the action *S* and seeking extrema leads to the stochastic Euler–Poincarè equation, and (2.4) is replaced by

2.19 $$d\frac{\delta \ell }{\delta \text{u}}+{\mathrm{ad}}_{\left(\text{u}\;dt+\sum_{i=1}^{m}\xi_{i}\circ \;dW_{i}\right)}^{*}\frac{\delta \ell }{\delta \text{u}}=a⋄\frac{\delta \ell }{\delta a}\;dt.$$

This equation no longer conserves energy, but it still has a Kelvin circulation theorem

2.20 $$d\oint_{C(t)}\frac{1}{\rho }\frac{\delta \ell }{\delta \text{u}}\cdot d\text{x}=\oint_{C(t)}a⋄\frac{\delta \ell }{\delta a}\cdot d\text{x}\;dt,$$

where points on the closed curve *C*(*t*) now follow the stochastic vector field in (2.18). A number of example stochastic geophysical fluid dynamics models derived in this framework were explored in [2].

Returning to our example of the incompressible Euler equation, the stochastic Euler–Poincaré equation with advected quantities becomes

2.21 $$\begin{array}{rl} & d(\rho \text{u})+\nabla \cdot (d\text{x}⊗\rho \text{u})+{\left(\nabla \;d\text{x}\right)}^{T}\cdot \rho \text{u}=\rho \nabla \left(-p+\frac{|\text{u}|}{2}\right)dt,\\ & d\rho +\nabla \cdot (\rho \;d\text{x})=0\\ \text{and}\; & \rho =\rho_{0}, \end{array}\}$$

where

2.22 $$d\text{x}=\text{u}\;dt+\sum_{i=1}^{m}\xi_{i}\circ \;dW_{i}.$$

For constant density, system (2.21) simplifies to the stochastic Euler fluid equations analysed in [6]; namely,

2.23 $$d\text{u}+d\text{x}\cdot \nabla \text{u}+{\left(\nabla \sum_{i=1}^{m}\xi_{i}\circ \;dW_{i}\right)}^{T}\cdot \text{u}=-\nabla p\;dt,\;\text{with}\;\nabla \cdot \text{u}=0.$$

*Aim of the paper*. The aim of this paper is to establish conditions under which the stochastic vector field Ansatz in equation (2.18) may be derived by applying the method of homogenization [13,7], for the purpose of gaining insight into the situations where such a model can be used in fluid dynamics.

## 3. Stochastic Lagrangian multi-time dynamics

We postulate a slow–fast evolutionary fluid flow map as the composition of a mean flow ${\bar{g}}_{t}$ depending on slow time *t* and a rapidly fluctuating flow $g_{t/\varepsilon }^{\prime }$ associated with the evolution of the fast time scales *t*/*ε*, with *ε*≪1. We define the flow map associated with the fast scales as the (spatially) smooth invertible map with smooth inverse (i.e. a diffeomorphism, or diffeo for short) given by the sum,

3.1 $$g_{t/\varepsilon }^{\prime }=\text{Id}+\zeta_{t/\varepsilon }\;\text{where}\;\varepsilon \ll 1.$$

The full flow map is taken to be the composition of ${\bar{g}}_{t}$ and $g_{t/\varepsilon }^{\prime }$, as

3.2 $$g_{t}=g_{t/\varepsilon }^{\prime }\circ {\bar{g}}_{t}={\bar{g}}_{t}+\zeta_{t/\varepsilon }\circ {\bar{g}}_{t}.$$

The Lagrangian trajectory of a fluid parcel is then given by **q**(**l**,*t*)=*g*_*t*_**l**, so that

3.3 $$\text{q}(\text{l},t)=\bar{\text{q}}(\text{l},t)+\zeta_{t/\varepsilon }\circ \bar{\text{q}}(\text{l},t),$$

where the vector **l** denotes the fluid label, e.g. the initial condition of the particles. The rapidly fluctuating vector displacement field

3.4 $$\zeta (\bar{\text{q}}(\text{l},t),t/\varepsilon ):=\zeta_{t/\varepsilon }\circ \bar{\text{q}}(\text{l},t)$$

is defined along the slow large-scale resolved trajectory, $\bar{\text{q}}$. At this point, (3.3) may be taken as exact, since it follows directly from the definition of the map *ζ*_*t*/*ε*_ in (3.1).

The tangent to the flow map *g*_*t*_ in (3.2) at **q**(**l**,*t*) along the Lagrangian trajectory (3.3) defines the Eulerian velocity vector field **u**, written as:

3.5 $${\dot{g}}_{t}\text{l}=\dot{\text{q}}(\text{l},t)=\text{u}(\text{q}(\text{l},t),t).$$

Differentiation of the Lagrangian trajectory (3.3) with the assumed fluctuating displacement field (3.4) yields

3.6 $$\text{u}(\bar{\text{q}}+\zeta_{t/\varepsilon }\circ \bar{\text{q}},t)=\dot{\bar{\text{q}}}+(\dot{\bar{\text{q}}}\cdot \nabla_{\bar{\text{q}}})\zeta (\bar{\text{q}}(\text{l},t),t/\varepsilon )+\frac{1}{\varepsilon }\partial_{t}\zeta .$$

We shall express the temporal partial derivative ∂_*t*_***ζ*** of the fluctuating vector displacement field ***ζ*** in (3.4) in terms of its empirical orthogonal eigenfunctions ***ϕ***_*i*_, *i*=1,2,…,*M*. We assume that the eigenfunctions ***ϕ***_*i*_ are slowly varying in space and are conditioned on the large-scale mean flow dynamics, so that we may write

3.7 $$\partial_{t}\zeta (\bar{\text{q}}(\text{l},t),t/\varepsilon )=\sum_{i=1}^{M}\lambda_{i}\left(\frac{t}{\varepsilon }\right)\phi_{i}(\bar{\text{q}}(\text{l},t)).$$

Note that the eigenvalues *λ*_*i*_(*t*) have temporal mean zero. We assume chaotic deterministic dynamics of the fast fluctuating *λ*_*i*_(*t*/*ε*) with

3.8 $${\dot{\lambda }}_{i}=\frac{1}{\varepsilon^{2}}h_{i}(\lambda ).$$

For convenience, let us summarize the deterministic multi-scale system describing the dynamics of the Lagrangian mean position variable $\bar{\text{q}}$, as follows:

3.9 $$\begin{array}{rl} (\mathrm{Id}+\nabla_{\bar{\text{q}}}\zeta )\dot{\bar{\text{q}}} & \;=\text{u}(\bar{\text{q}}+\zeta_{t/\varepsilon }\circ \bar{\text{q}},t)-\frac{1}{\varepsilon }\partial_{t}\zeta (\lambda ,\bar{\text{q}}(\text{l},t)), \end{array}$$

3.10 $$\begin{array}{rl} {\dot{\lambda }}_{i} & \;=\frac{1}{\varepsilon^{2}}h_{i}(\lambda ) \end{array}$$

3.11 $$\begin{array}{rl} \text{and}\;\partial_{t}\zeta & \;=\sum_{i=1}^{M}\lambda_{i}\left(\frac{t}{\varepsilon }\right)\phi_{i}(\bar{\text{q}}(\text{l},t)). \end{array}$$

The next section employs homogenization theory to show how this set of equations converges for *ε*→0 to solutions of a certain stochastic Lagrangian dynamics on long time scales.

## 4. Diffusive limit of stochastic Lagrangian dynamics

Homogenization is a mathematical tool for extracting the statistical behaviour of the slow degrees of freedom in a multi-scale system, by providing an effective stochastic differential equation for the slow degrees of freedom [13,7]. Homogenization represents the integrated effect of the fast (either stochastic, or chaotic) dynamics on the slow variables as noise. Homogenization was initially developed for stochastic multi-scale systems [14–16]. However, it has been extended recently to apply for deterministic multi-scale systems with sufficiently chaotic fast dynamics [17–19]. In particular, homogenization applies to deterministic multi-scale systems for slow variables $x\in R^{d}$ and fast variables $y\in R^{m}$ of the form,

4.1 $$\dot{x}=\;\;\;\frac{1}{\varepsilon }\;f_{0}(x,y)+f_{1}(x,y)$$

and

4.2 $$\dot{y}=\;\;\;\frac{1}{\varepsilon^{2}}\;g_{0}(y).$$

We assume that the vector fields $f_{0}:R^{d}\times R^{m}\to R^{d}$, $f_{1}:R^{d}\times R^{m}\to R^{d}$ and $g:R^{m}\to R^{m}$ satisfy certain regularity conditions. We consider sufficiently chaotic fast *y*-dynamics with a compact chaotic attractor $\Lambda \subset R^{m}$ and ergodic invariant probability measure *μ*. Homogenization requires the so-called *centring condition* $\int_{\Lambda }f_{0}\;d\mu =0$, i.e. that averaging would result in trivial dynamics associated with *f*_0_. It is instructive to write the slow dynamics in the integrated form

4.3 $$x(t)=x(0)+\varepsilon^{2}\int_{0}^{t/\varepsilon^{2}}f_{1}(x(\tau ),y(\tau ))\;d\tau +\varepsilon \int_{0}^{t/\varepsilon^{2}}f_{0}(x(\tau ),y(\tau ))\;d\tau ,$$

where we perform the time integral over the fast time scale *τ*=*t*/*ε*^2^. The first term involving *f*_1_ approaches for *ε*→0 its ergodic mean by means of the law of large numbers. The second term involves summing up sufficiently decorrelated variables *f*_0_ with mean zero (the centring condition) and in the limit *ε*→0 leads to Brownian noise by means of the central limit theorem. The randomness comes here from the initial conditions of the fast ergodic deterministic dynamical system. Under the above hypotheses this heuristic argument can be made rigorous [17–19]: as *ε*→0 the slow dynamics *x*(*t*) converges weakly in the space of continuous functions $C([0,T],R^{d})$ for some *T*>0 to the solution of the homogenized Itô stochastic differential equation

4.4 $$dX=F(X)\;dt+\Sigma (X)\;dW_{t},$$

where *W*_*t*_ denotes Brownian motion and the drift coefficient is given by

4.5 $$F(x)=\int f_{1}(x,y)\;\mu (dy)+\int_{0}^{\infty }ds\int f_{0}(x,y)\cdot \nabla f_{0}(x,y(s))\mu (dy)$$

and the diffusion coefficient is defined by

4.6 $$\Sigma (X)\Sigma^{T}(X)=\int_{0}^{\infty }ds\int (f_{0}(y)⊗f_{0}(y(s))+f_{0}(y(s))⊗f_{0}(y))\mu (dy),$$

where the outer product between two vectors is defined as (*a*⊗*b*)_*ij*_=*a*_*i*_*b*_*j*_ [19–21].

We remark that, in one spatial dimension *d*=1, the drift term reduces to

$$F(X)=\int f_{1}(x,y)\mu (dy)+\frac{1}{2}\Sigma (X)\Sigma^{T}(X),$$

which implies that the noise is of Stratonovich type. In higher dimensions, the noise is neither of Stratonovich nor of the Itô type, and the drift term contains non-trivial corrections stemming from *f*_0_. The required chaoticity assumption needed to assure the functional central limit theorem, the so-called *weak invariance principle*, is rather innocuous, and it allows convergence to diffusive laws to be proven for a large class of chaotic dynamical systems. In particular, no assumption about mixing is needed. For deterministic maps, the convergence to Brownian motion holds when the correlation function of *f*_0_ is summable. For flows, it suffices that there is a Poincaré map with these properties (irrespective of the mixing properties of the flow). These systems include, but extend far beyond, Axiom A diffeomorphisms and flows, Hénon-like attractors and Lorenz attractors. Precise statements about their validity can be found in [22–24]. We remark that for weakly chaotic dynamics, when the correlations are not summable/integrable, e.g. for systems exhibiting intermittency with long laminar periods, the central limit theorem breaks down and the noise is not Brownian anymore. Instead, the noise is *α*-stable, allowing for unbounded variance and jumps [18].^1^

We now show that the deterministic Lagrangian multi-scale system (3.9)–(3.11) developed in the previous section is amenable to homogenization and we will derive the associated effective stochastic limit system. For these purposes, we assume sufficiently chaotic dynamics for *λ*_*i*_ with ergodic measure *μ*(*dλ*).

First, we show that the centring condition is satisfied. Denoting by angular brackets the average over the fast measure *μ*(*dλ*), we express the centring condition as

4.7 $$\begin{array}{rl} \left\langle {\left(\mathrm{Id}+\nabla \zeta \right)}^{-1}\partial_{t}\zeta \right\rangle & \;=\langle {\left(\nabla g_{t/\varepsilon }^{\prime }\right)}^{-1}\partial_{t}g_{t/\varepsilon }^{\prime }\rangle \\ & \;=\langle (\partial_{t}({\left(g_{t/\varepsilon }^{\prime }\right)}^{-1}))\circ g_{t/\varepsilon }^{\prime }\rangle =0. \end{array}$$

Consequently, the mean displacements in the fluctuating map vanish, when pulled back to (i.e. transformed back to vectors relative to) the mean Eulerian coordinates. In fact, this condition simply defines how we take the mean; namely, it is defined so that this condition is satisfied.

Homogenization theory for deterministic multi-scale systems as developed in [17–19] assures that the slow dynamics of the deterministic multi-scale Lagrangian particle system (3.9)–(3.11) is on long time scales described by the stochastic differential equation

4.8 $$d\bar{\text{Q}}=\bar{\text{U}}(\bar{\text{Q}})\;dt+\sigma (\bar{\text{Q}})\;dW_{t},$$

where the drift term is given by

4.9 $$\bar{\text{U}}(\bar{\text{Q}})=\langle {\left(\mathrm{Id}+\nabla \zeta \right)}^{-1}\text{u}(\bar{\text{Q}}(\text{l},t)+\zeta ,t)\rangle +\langle \partial_{t}\zeta \nabla_{\bar{\text{Q}}}\partial_{t}\zeta \rangle$$

and the diffusion tensor is given by

4.10 $$\frac{1}{2}\sigma \sigma^{T}=\int_{0}^{\infty }ds\sum_{i,j=1}^{M}\langle \lambda_{i}(0)\lambda_{j}(s){\left(\mathrm{Id}+\nabla \zeta \right)}^{-1}P_{ij}\;{\left(\mathrm{Id}+\nabla \zeta \right)}^{-T}\rangle ,$$

where $P_{ij}=\phi_{i}(\bar{\text{q}}(\text{l},t))\phi_{j}{\left(\bar{\text{q}}(\text{l},t)\right)}^{T}$, and d**W**_*t*_=(d*W*_1_,…,d*W*_*M*_) is a vector of independent Brownian motions (cf. (4.5) and (4.6)). Upon defining ***ξ***_*i*_ as the *i*th row of *σ*, this result matches the Ansatz (2.18) introduced in the stochastic variational framework [2,6].

Note that because of the multiplicative nature of the noise, the drift (4.9) is not just given by the average mean flow $\left\langle {\left(\mathrm{Id}+\nabla \zeta \right)}^{-1}\text{u}(\bar{\text{Q}}(\text{l},t)+\zeta ,t)\right\rangle$; there is an additional drift component provided by the fluctuating vector displacement field.

## 5. Summary and discussion

The diffusive limit we have described relies on homogenization theory. In order to apply it we had to make several assumptions. First, we made an *envelope assumption*, that the fluctuating vector displacement field ***ζ*** varies rapidly in time, but only slowly in space. Second, we made a *chaoticity assumption*, whereby the temporal derivative of ***ζ*** evolves according to some unspecified chaotic dynamics. The microscopic details of the fast chaotic dynamics are not relevant for the emergence of stochastic Lagrangian particle dynamics, but they do feature in the diffusion tensor in equation (4.10). In order to build reliable stochastic coarse-grained fluid models, one needs to determine the drift and diffusion terms (4.9) and (4.10), respectively. The advantage of the approach taken in [2,6] and in this paper is that the fluctuating small-scale field ***ζ*** is Eulerian. One can, therefore, obtain the statistics by investigating time series of Eulerian flow fields in terms of their empirical orthogonal eigenfunctions ***ϕ*** and their temporal evolution. The data may be generated either by analysis of numerical high-resolution simulations, or from observational data.

The diffusive limit in the Lagrangian dynamics for the slowly varying mean flow then forms the basis for the variational approach proposed in [2,6] to derive stochastic partial differential equations for fluid systems. The same procedure can, however, also be applied to give a deterministic multi-scale backbone for the stochastic fluid equations derived in [1,3–5].

Equation (4.9) shows that there are two contributions to the mean velocity $\bar{\text{u}}$. The first term arises from averaging the full velocity over the fluctuations, and the second term arises purely from the structure of the statistics of the time derivative of the fluctuation vector ***ζ***. The second term shows that the fluctuating dynamics affects the mean flow. Thus, beyond being potentially useful as a means of uncertainty quantification, the sum of vector fields in (2.18) in the conditions treated here represents a *bona fide* decomposition of the fluid transport velocity into a mean plus fluctuating flow.

The present derivation shows that the stochastic fluid equations can be regarded as modelling the evolution of the mean velocity $\bar{\text{u}}$ for a fluid that has a fluctuating velocity that is O(1/ϵ). These equations also provide a possible way to calculate the EOF eigenvectors *ξ*_*i*_ describing spatial correlations for the stochastic model proposed in [2,6]. For this purpose, one should compute a large number of Lagrangian trajectories, then apply a low-pass spatial filter to them, and compute time series of displacement vectors ***ζ***_**t**_ for each trajectory. The values of ***ζ***_**t**_ may be binned according to coarse-grained grid boxes, and then combined in the formula for *σ*. One can then compute EOFs of *σ*, which, in turn, yield the EOF eigenvectors *ξ*_*i*_ for the stochastic model proposed in [2,6]. The performance of the stochastic model constructed this way is currently being evaluated, using data assimilation algorithms.

We conclude by stating that real atmospheric and oceanic flows do not exhibit the limit of infinite time-scale separation required by homogenization theory. Finite time-scale effects amount to finite sampling effects in the derivation of the central limit theorem (see the last term in (4.3)). These can be calculated in the form of Edgeworth approximations and involve integrals over higher-order autocorrelation functions [26].

## Footnotes

1 We use the terminology strongly and weakly chaotic here in a manner different from the usual distinction between exponential and algebraic decay of correlations; cf. [25] for further discussion of this difference.

## Data accessibility

This paper has no additional data.
